# Correlation of NICU anthropometry in extremely preterm infants with brain development and language scores at early school age

**DOI:** 10.1038/s41598-023-42281-0

**Published:** 2023-09-15

**Authors:** Ting Ting Fu, Maria E. Barnes-Davis, Hisako Fujiwara, Alonzo T. Folger, Stephanie L. Merhar, Darren S. Kadis, Brenda B. Poindexter, Nehal A. Parikh

**Affiliations:** 1 Division of Neonatology, Perinatal Institute, Cincinnati Children’s Hospital Medical Center, 3333 Burnet Avenue, MLC 7009, Cincinnati, OH 45229-3026 USA; 2 Department of Pediatrics, University of Cincinnati College of Medicine, Cincinnati, OH USA; 3 Division of Neurology, Cincinnati Children’s Hospital Medical Center, Cincinnati, OH USA; 4 Division of Biostatistics and Epidemiology, Cincinnati Children’s Hospital Medical Center, Cincinnati, OH USA; 5 Neurosciences and Mental Health, Hospital for Sick Children, Toronto, ON Canada; 6 Department of Physiology, University of Toronto, Toronto, ON Canada; 7 Division of Neonatology, Department of Pediatrics, Emory University School of Medicine and Children’s Healthcare of Atlanta, Atlanta, GA USA

**Keywords:** Paediatrics, Magnetic resonance imaging

## Abstract

Growth in preterm infants in the neonatal intensive care unit (NICU) is associated with increased global and regional brain volumes at term, and increased postnatal linear growth is associated with higher language scores at age 2. It is unknown whether these relationships persist to school age or if an association between growth and cortical metrics exists. Using regression analyses, we investigated relationships between the growth of 42 children born extremely preterm (< 28 weeks gestation) from their NICU hospitalization, standardized neurodevelopmental/language assessments at 2 and 4–6 years, and multiple neuroimaging biomarkers obtained from T1-weighted images at 4–6 years. We found length at birth and 36 weeks post-menstrual age had positive associations with language scores at 2 years in multivariable linear regression. No growth metric correlated with 4–6 year assessments. Weight and head circumference at 36 weeks post-menstrual age positively correlated with total brain volume and negatively with global cortical thickness at 4–6 years of age. Head circumference relationships remained significant after adjusting for age, sex, and socioeconomic status. Right temporal cortical thickness was related to receptive language at 4–6 years in the multivariable model. Results suggest growth in the NICU may have lasting effects on brain development in extremely preterm children.

## Introduction

Despite advancements in neonatal nutrition strategies, up to 50% of very low birth weight infants plot at less than 10th percentile for their corrected age on the weight curve at time of discharge from the neonatal intensive care unit (NICU)^1–3^. Optimizing nutrition to promote growth during NICU hospitalization is a key area of research and clinical interest as greater gains in somatic anthropometric measurements (weight, length, head circumference, body mass index (BMI), and lean mass) have all been linked to better neurodevelopmental outcomes in early childhood, including decreased incidence of cerebral palsy, improved cognitive, motor, and language scores, and higher intelligence quotients^4–10^. In addition, weight, BMI, lean mass, and increased early macronutrient intake in preterm infants are associated with improved brain global and regional volumes and cross-sectional diameters on magnetic resonance imaging (MRI) at term equivalent age^11–13^. Lower gestational age is also associated with smaller brain volumes in preterm infants and greater risk for neurodevelopmental impairment^14^. However, it is not known if somatic growth in the NICU is associated with brain volumes and neurodevelopmental outcomes at early school age.

The relationship between NICU somatic growth and cortical metrics also has not been examined. Cortical thickness is considered to be a measurement of the radial columns of pyramidal cells and interneurons that comprise the six layers of the cerebral cortex^15^. It increases as neuronal populations grow prenatally and postnatally, then starts to subside due to pruning of synapses and efficiency of brain networks during childhood^16–18^. However, the relationship of cortical thickness and outcomes in premature infants is not robust. One group did not find significant regional differences in cortical thickness between preterm and term children^19^. Some studies report increased cortical thickness in preterm children versus term children^20–24^, while others report decreased cortical thickness in preterm children^20,21,25,26^.

Furthermore, preterm infants with poor linear growth specifically during their NICU course have demonstrated worse language scores at age 2^5,6^. While direct head growth can be noted by occipitofrontal circumference, linear growth is also thought to be a proxy for protein and lean mass accretion and, consequently, organ development and brain growth^27–29^. In our pilot study of extremely preterm (EPT, born less than 28 weeks gestation) children at 4–6 years, increased cortical thickness in right temporal areas and structural connectivity in a bitemporal pathway involving the cerebellum were positively correlated with language scores at the same age^24,30^. Here we evaluate the relationship of postnatal NICU growth, a potentially modifiable factor, with brain and language outcomes in this cohort.

## Methods

### Participants

This is an analysis of 42 EPT children who were previously recruited for a larger ongoing multimodal neuroimaging investigation examining brain-based markers of language development in EPT children and their term counterparts. Children were recruited from Cincinnati-area NICUs if they were born at < 28 weeks gestation in the years 2009–2012 and had no grade 3–4 intraventricular hemorrhage or periventricular leukomalacia on neonatal cranial ultrasound. Medical chart reviews were performed for EPT participants to verify clinical data (gestational age, cranial ultrasound results) obtained from parents. Children with cerebral palsy, seizures, migraines, history of learning or speech disability, or history of neurologic disorder were excluded. Full details of the parent study, including family surveys of demographic data, have been published elsewhere^24,30–33^. The study was approved by the Cincinnati Children’s Hospital Institutional Review Board and conforms to the United States Federal Policy for the Protection of Human Subjects. Written informed consent was obtained from parents. Methods were carried out in accordance with the Declaration of Helsinki.

### Neuroimaging acquisition and analysis

Three-dimensional T1-weighted MRI was obtained for each subject on a 3.0 T Phillips Achieva scanner with a T1 turbo field echo sequence (TR/TE = 8.055/3.68 ms, 1.0 × 1.0 × 1.0 mm voxels, Matrix = 256 × 256 × 160). All scans were read by a board-certified radiologist with certificates of added qualifications in pediatric radiology and neuroradiology who was blinded to clinical history. Images were subjected to volumetric and cortical thickness analyses on a whole-brain level using FreeSurfer (Version 6.0.0 http://surfer.nmr.mgh.harvard.edu/ running in Mac OSX operating system version 10.12.6). As we and others have described previously, an automated pipeline in FreeSurfer was used for cortical reconstruction, segmentation, and non-linear surface-based registration^24,34–37^. Cortical thickness was calculated as the closest distance from the gray/white boundary to the gray/CSF boundary per vertex^34^. Regional volumes and cortical thickness measurements were normalized for estimated total intracranial volume (eTIV) by dividing each value by eTIV, then multiplying by 10^6^ for scale. Mean cortical thickness and brain volumetrics were calculated by hemisphere and by region.

### Infant data from NICU hospitalization

Weight, length, and head circumference (HC) at birth and at 36 weeks post-menstrual age (PMA) from NICU were obtained by medical chart review. For infants discharged prior to 36 weeks PMA, their discharge measurements were used. For each measurement, z-scores accounting for PMA and sex were calculated using the Fenton reference growth curve^38^. Changes in z-score were defined as the difference from birth to 36 weeks PMA for each anthropometric z-score. BMI was calculated per the Olsen intrauterine reference^39^.

Incidences of common comorbidities were also recorded. Severe bronchopulmonary dysplasia (BPD) was defined as needing greater than 2 L/min of nasal cannula support at 36 weeks PMA (grade 2 or higher)^40^; severe retinopathy of prematurity (ROP) was defined as ROP requiring treatment (either surgery or intravitreal injection of anti-vascular endothelial growth factor agents) or evidence of retinal detachment. Cases of necrotizing enterocolitis (NEC) included stage 2 and stage 3 NEC per modified Bell’s criteria, and late onset sepsis was defined as having a positive blood culture after the first 72 h of life.

### Neuropsychological assessments

Children underwent assessment with the Peabody Picture Vocabulary Test 4th Edition (PPVT4)^41^; Expressive Vocabulary Test 2nd edition (EVT2)^42^; and Wechsler Nonverbal Scale of Ability (WNV)^43^, and age-standardized scores were reported. The EVT2 and PPVT4 were used to assess expressive and receptive vocabulary, respectively. They were chosen for their brevity and their high correlation with verbal intelligence, especially in children^44,45^. They are also widely used in studies of prematurity, including our prior work^24,30–33,46–48^. Infants had the Bayley Scales of Infant Development 3rd edition (BSID-III)^49^ performed at 22–26 months corrected age by a certified examiner as part of our infant follow-up clinic protocol. These results were obtained from medical chart review.

### Statistical analysis

Pearson correlations were first performed to screen for linear relationships. Analyses were completed using each anthropometric z-score and BMI at birth or 36 weeks PMA or the change between those time points as the predictor. Multiple univariate regression analyses were performed using analysis of maximum likelihood. Dependent outcomes of interest were BSID-III scores at 2 years (cognitive, language, and motor composites), neuropsychological assessments at 4–6 years (PPVT4, EVT2, WNV), global brain volume (eTIV), global cortical thickness (left and right hemispheres), regional brain volumes (corpus callosum, left and right cerebellum, left and right temporal), and regional cortical thickness (left and right temporal). Both normalized and non-normalized brain metrics were evaluated. The relationships between brain metrics and PPVT4/EVT2/WNV assessments as well as between BSID-III and PPVT4/EVT2/WNV scores were also assessed. We did not account for multiple comparisons given the pilot nature of this study and to reduce the likelihood for type II error^50^.

For the multivariable linear regression models, five covariates of interest were selected a priori: gestational age (GA); sex; highest parental education reported at time of assessment; family income reported at time of assessment; and corrected age at time of assessment/MRI^51^. These measures were selected as potential confounding factors to adjust for in the multivariable model. Covariates were included in models if there was evidence of potential confounding, based on significance observed in univariate analyses (Supplementary Table 1) or assessing whether the parameter estimate changed by at least 10% with or without inclusion of each covariate (not shown). Furthermore, for each growth metric, multicollinearity was assessed with our covariates using a variance inflation factor threshold of 10, and no collinearity was detected.

A table depicting which covariates were included in each multivariable model is shown in Supplementary Table 2. All models adjusted for GA, sex, highest parental education, and family income (insurance status used as a proxy for models evaluating BSID-III). Regression models with neuroimaging markers included corrected at age MRI. Again, due to the pilot nature of our analysis, we did not include additional corrections for multiple comparisons at this step. Missing data was not imputed for each analysis. Statistical analyses were completed using SAS software version 9.4 (SAS Institute Inc., Cary, NC). Results were considered statistically significant for p < 0.05.

## Results

Table 1 shows the patient demographics and NICU clinical outcomes of the 42 EPT children, of which 33 had high quality brain imaging sufficient for analysis. Table 2 presents the results of their neurodevelopmental testing and family demographics at the time of each assessment. 37 had completed BSID-III scores. One participant was unable to complete PPVT4 testing, and a different participant was unable to complete WNV testing.

**Table 1:** Participant characteristics and neonatal outcomes.

	(n = 42)
Demographics	
Male	21 (50%)
Gestational age at birth (weeks)	26.3 (24.9–26.9)
Birth weight (g)	850.2 ± 124.2
Gestational age at discharge (weeks)	39.4 ± 3.5
NICU anthropometric z-scores	
Birth	
Weight	0.2 ± 0.8
Length	0.2 ± 0.8
Head circumference	0.2 ± 0.9
36 Weeks post-menstrual age	
Weight	− 0.6 ± 0.7
Length	− 1.6 ± 0.8
Head circumference	− 0.7 ± 0.7
Change in z-score	
Weight	− 0.8 ± 0.7
Length	− 1.8 ± 0.8
Head circumference	− 0.9 ± 0.9
NICU comorbidities	
Severe bronchopulmonary dysplasia	5 (11.9%)
Severe retinopathy of prematurity	2 (4.8%)
Necrotizing enterocolitis	5 (11.9%)
Late onset sepsis	4 (9.5%)

Mean ± SD, median (IQR), or n (%).

**Table 2:** Participant assessment results and socioeconomic characteristics at follow-up.

BSID-III testing at 2 years (n = 37)	
Age at assessment (years)	2.2 ± 0.2
Cognitive composite score	105.8 ± 15.8
Language composite score	94.7 ± 15.3
Motor composite score	102.5 ± 10.9
Highest parental education	
Some high school	2 (5.4%)
High school	10 (27.0%)
Some college	6 (16.2%)
College	12 (32.4%)
Graduate level	7 (18.9%)
Insurance	
Public only	15 (40.5%)
Private or both	22 (59.5%)
Assessments at 4–6 years (n = 42)	
Age at assessment/MRI (years)	5.5 ± 0.9
PPVT4 (receptive language) (n = 41)	111.6 ± 11.3
EVT2 (expressive language)	100.9 ± 9.6
WNV (general abilities) (n = 41)	102.7 ± 13.5
Highest parental education	
High school	6 (14.6%)
Some college	14 (34.1%)
College	8 (19.5%)
Some graduate level	3 (7.3%)
Graduate level	10 (24.4%)
Family income at 4–6 years (n = 41)	
Less than $50,000	15 (36.6%)
Greater than $50,000	26 (63.4%)

Mean ± SD or n (%).

### Growth and developmental outcomes

No NICU growth metric was associated with PPVT4, EVT2, or WNV language scores at 4–6 years in either correlation analysis or multivariable linear regression models.

With respect to BSID-III composite scores at 2 years, length z-score at 36 weeks PMA was strongly correlated with language (r = 0.42, p = 0.009). Change in length z-score was also positively associated with cognitive composite score (r = 0.36, p = 0.027), whereas change in BMI was negatively associated (r =  − 0.35, p = 0.035). Of these associations, only the relationship between length z-score at 36 weeks PMA and language remained significant after controlling for sex, GA, highest parental education, and insurance (Table 3). The multivariable model identified additional direct associations between birth length z-score with language composite score and between birth BMI with motor composite score (Table 3).

**Table 3:** Multivariable regression model comparing growth with BSID-III assessments.

		BSID-III		
		Cognitive composite	Language composite	Motor composite
Birth	Weight z-score	0.3 (− 7.8, 8.4)	3.3 (− 4.9, 11.4)	− 2.2 (− 9.0, 4.6)
	Length z-score	− 2.6 (− 8.7, 3.4)	**6.8 (1.0, 12.5)**	4.6 (− 0.3, 9.5)
	HC z-score	3.0 (− 2.9, 8.9)	0.04 (− 6.1, 6.2)	− 0.05 (− 5.1, 5.0)
	BMI	2.2 (− 4.2, 8.7)	− 4.2 (− 10.7, 2.2)	− **7.2**^**#**^** (**− **12.0,** − **2.4)**
36 Weeks PMA	Weight z-score	− 0.4 (− 7.3, 6.5)	2.7 (− 4.2, 9.7)	− 0.5 (− 6.4, 5.3)
	Length z-score	− 0.8 (− 7.9, 6.2)	**7.9 (1.3, 14.4)**	2.1 (− 3.9, 8.0)
	HC z-score	− 0.7 (− 6.7, 5.4)	3.6 (− 2.4, 9.7)	− 1.2 (− 6.3, 3.9)
	BMI	0.4 (− 3.1, 3.8)	− 1.8 (− 5.2, 1.7)	− 1.4 (− 4.3, 1.5)
Change from birth to 36 weeks PMA	Weight z-score	− 0.7 (− 8.1, 6.6)	0.4 (− 7.1, 7.9)	1.2 (− 5.0, 7.4)
	Length z-score	2.1 (− 4.1, 8.4)	− 0.8 (− 7.3, 5.6)	− 3.2 (− 8.4, 2.0)
	HC z-score	− 3.3 (− 8.9, 2.3)	3.3 (− 2.5, 9.0)	− 1.1 (− 5.9, 3.8)
	BMI	− 0.2 (− 3.6, 3.1)	− 0.5 (− 3.9, 2.9)	0.6 (− 2.2, 3.4)

Adjusted for sex, gestational age, highest parental education (at time of BSID assessment), insurance. Parameter estimates (95% CI) represent change in BSID-III composite scores per increment in growth (1.0 z-score or 1.0 kg/m^2^ in BMI). Statistically significant values with p < 0.05 are in bold.

^#^Denotes p < 0.01.

### Growth and neuroimaging markers

Pearson correlations comparing growth metrics with normalized global and regional brain MRI markers are shown in Table 4. The strongest correlation was HC z-score at 36 weeks PMA with eTIV (r = 0.45, p = 0.009). Several growth metrics at birth and 36 weeks PMA were negatively associated with normalized measurements, both global and regional, but this was not observed when utilizing non-normalized brain MRI measurements (Supplementary Table 3). Change in growth z-scores from birth to 36 weeks PMA was not associated with any global or regional brain metrics. In the multivariable model, after adjusting for all five covariates of interest, the relationship between HC z-score at 36 weeks PMA and eTIV remained significant, as did the negative associations with normalized left and right hemisphere cortical thickness (Table 5). Again, these negative parameter estimates were not noted when using non-normalized MRI measurements (Supplementary Table 4).

**Table 4:** Pearson correlations comparing growth with brain MRI metrics.

		Global brain metrics			Regional brain metrics						
		eTIV (mm^3^)	Cortical thickness (left) (mm)	Cortical thickness (right) (mm)	Volume: corpus callosum (mm^3^)	Volume: cerebellum (left) (mm^3^)	Volume: cerebellum (right) (mm^3^)	Volume: temporal (left) (mm^3^)	Volume: temporal (right) (mm^3^)	Cortical thickness: temporal (left) (mm)	Cortical thickness: temporal (right) (mm)
Birth	Weight z-score	0.29	− 0.32	− **0.35**	− 0.03	− 0.33*	− 0.33*	0.06	0.23	− 0.26	− 0.23
	Length z-score	0.14	− 0.11	− 0.13	− 0.08	− 0.20	− 0.21	0.23	**0.37**	− 0.08	0.01
	HC z-score	**0.38**	− **0.36**	− **0.38**	0.01	− 0.21	− 0.19	− 0.01	0.14	− 0.32	− 0.30
	BMI	0.34*	− **0.39**	− **0.41**	0.15	− 0.28	− 0.26	− 0.06	0.08	− 0.32	− 0.32
36 Weeks PMA	Weight z-score	**0.36**	− **0.39**	− **0.37**	− 0.04	− 0.34*	− 0.30	0.13	0.17	− 0.28	− 0.27
	Length z-score	0.14	− 0.08	− 0.07	− 0.01	− 0.07	− 0.12	0.28	0.34*	0.02	0.00
	HC z-score	**0.45**^**#**^	− **0.44**	− **0.41**	− 0.12	− 0.16	− 0.18	0.05	0.25	− **0.35**	− 0.32
	BMI	0.29	− **0.37**	− **0.36**	0.01	− 0.31	− 0.24	− 0.07	− 0.09	− 0.33*	− 0.31
Change from birth to 36 weeks PMA	Weight z-score	0.05	− 0.05	0.00	− 0.01	0.02	0.05	0.07	− 0.08	− 0.01	− 0.03
	Length z-score	0.00	0.03	0.05	0.07	0.11	0.08	0.07	− 0.01	0.10	0.00
	HC z-score	− 0.03	0.01	0.06	− 0.12	0.09	0.05	0.06	0.07	0.05	0.05
	BMI	0.13	− 0.19	− 0.17	− 0.06	− 0.18	− 0.12	− 0.04	− 0.12	− 0.18	− 0.16

All brain metrics normalized for eTIV.

Statistically significant values with p < 0.05 in bold.

^#^Denotes p < 0.01.

*Denotes p = 0.05–0.06.

**Table 5:** Multivariable regression model comparing growth with brain MRI metrics.

		Global brain metrics			Regional brain metrics						
		eTIV (mm^3^)	Cortical thickness (left) (mm)	Cortical thickness (right) (mm)	Volume: corpus callosum (mm^3^)	Volume: cerebellum (left) (mm^3^)	Volume: cerebellum (right) (mm^3^)	Volume: temporal (left) (mm^3^)	Volume: temporal (right) (mm^3^)	Cortical thickness: temporal (left) (mm)	Cortical thickness: temporal (right) (mm)
Birth	Weight z-score	1277.0 (− 68,369.4, 70,923.5)	0.002 (− 0.12, 0.12)	− 0.004 (− 0.11, 0.10)	188.5 (− 8.3, 385.3)	− 2410.4* (− 4851.2, 30.4)	− 2401.6 (− 5106.7, 303.6)	422.6 (− 1278.7, 2123.9)	261.1 (− 1369.3, 1891.5)	0.02 (− 0.1, 0.15)	0.02 (− 0.11, 0.15)
	Length z-score	− 30,938.6 (− 97,103.2, 35,226.0)	0.06 (− 0.05, 0.17)	0.06 (− 0.04, 0.16)	98.1 (− 102.9, 299.1)	− 1602.1 (− 4068.6, 864.5)	− 1607.9 (− 4318.7, 1102.8)	1199.5 (− 378.4, 2777.4)	1045.3 (− 474.8, 2565.5)	0.07 (− 0.04, 0.19)	0.11 (− 0.01, 0.23)
	HC z-score	32,433.4 (− 21,691.8, 86,558.6)	− 0.04 (− 0.13, 0.05)	− 0.04 (− 0.12, 0.05)	121.8 (− 40.2, 283.8)	− 1185.2 (− 3241.7, 871.3)	− 1151.9 (− 3412.7, 1108.9)	− 377.7 (− 1738.8, 983.5)	− 289.5 (− 1593.0, 1013.9)	− 0.03 (− 0.13, 0.07)	− 0.04 (− 0.14, 0.07)
	BMI	40,060.5 (− 23,134.7, 103,255.6)	− 0.07 (− 0.17, 0.04)	− 0.07 (− 0.17, 0.03)	123.1 (− 69.1, 315.3)	− 1617.4 (− 4001.8, 767.1)	− 1481.6 (− 4118.1, 1155.0)	− 485.3 (− 2077.9, 1107.4)	− 295.2 (− 1824.0, 1233.6)	− 0.05 (− 0.17, 0.06)	− 0.07 (− 0.19, 0.05)
36 Weeks PMA	Weight z-score	48,112.8 (− 14,268.7, 110,494.3)	− 0.07 (− 0.17, 0.04)	− 0.06 (− 0.16, 0.04)	75.0 (− 121.9, 272.0)	− 1782.1 (− 4152.6, 588.4)	− 1472.8 (− 4117.3, 1171.7)	117.4 (− 1491.4, 1726.3)	264.3 (− 1269.5, 1798)	− 0.05 (− 0.16, 0.07)	− 0.04 (− 0.16, 0.09)
	Length z-score	− 2657.8 (− 65,378.8, 60,063.2)	0.002 (− 0.10, 0.11)	0.004 (− 0.09, 0.10)	34.3 (− 156.0, 224.7)	− 1609.5 (− 3890.6, 671.7)	− 1487.3 (− 4010.6, 1036.0)	746.6 (− 762.1, 2255.2)	736.6 (− 702.6, 2175.8)	0.03 (− 0.08, 0.14)	0.03 (− 0.09, 0.15)
	HC z-score	**66,838.1 (10,423.4, 123,252.8)**	− **0.11 (**− **0.21,** − **0.02)**	− **0.09 (**− **0.18,** − **0.004)**	− 13.5 (− 204.9, 178)	− 1036.3 (− 3383.2, 1310.5)	− 889.0 (− 3470.2, 1692.2)	− 118.3 (− 1663.5, 1426.8)	558.2 (− 900.2, 2016.5)	− 0.09 (− 0.19, 0.01)	− 0.08 (− 0.19, 0.04)
	BMI	26,358.9 (− 3320.6, 56,038.4)	− 0.03 (− 0.09, 0.02)	− 0.03 (− 0.08, 0.02)	44.0 (− 50.5, 138.6)	− 231.7 (− 1427.9, 964.6)	− 97.5 (− 1408.4, 1213.3)	− 206.9 (− 979.6, 565.7)	− 137.9 (− 878.4, 602.6)	− 0.04 (− 0.09, 0.02)	− 0.03 (− 0.09, 0.02)
Change from birth to 36 weeks PMA	Weight z-score	45,045.8 (− 16,308.3, 106,399.8)	− 0.06 (− 0.17, 0.04)	− 0.05 (− 0.15, 0.04)	− 88.1 (− 279.9, 103.7)	337.9 (− 2091.0, 2766.7)	626.9 (− 2018.7, 3272.6)	− 246.2 (− 1818.9, 1326.5)	31.7 (− 1473.9, 1537.3)	− 0.06 (− 0.17, 0.05)	− 0.05 (− 0.17, 0.07)
	Length z-score	32,029.7 (− 38,993.4, 103,052.8)	− 0.07 (− 0.19, 0.05)	− 0.07 (− 0.17, 0.04)	− 67.1 (− 285.3, 151.0)	− 295.1 (− 3032.3, 2442.0)	− 126.2 (− 3119.8, 2867.3)	− 387.6 (− 2155.1, 1379.9)	− 223.7 (− 1916.7, 1469.4)	− 0.04 (− 0.16, 0.09)	− 0.08 (− 0.21, 0.06)
	HC z-score	18,932.4 (− 34,669.3, 72,534.1)	− 0.04 (− 0.13, 0.04)	− 0.03 (− 0.12, 0.05)	− 124.8 (− 281.2, 31.6)	349.8 (− 1699.1, 2398.7)	427.6 (− 1809.9, 2665.0)	268.5 (− 1057.4, 1594.4)	686.5 (− 551.8, 1924.9)	− 0.04 (− 0.13, 0.06)	− 0.02 (− 0.12, 0.08)
	BMI	13,138.6 (− 14,174.6, 40,451.8)	− 0.01 (− 0.06, 0.03)	− 0.01 (− 0.05, 0.03)	11.7 (− 72.9, 96.4)	114.3 (− 940.8, 1169.4)	193.5 (− 957.8, 1344.8)	− 72.1 (− 755.7, 611.4)	− 53.2 (− 706.4, 600.1)	− 0.02 (− 0.07, 0.03)	− 0.01 (− 0.07, 0.04)

Adjusted for sex, gestational age, highest parental education, family income, age at MRI. All brain metrics normalized for eTIV.

Parameter estimates (95% CI) represent change in brain MRI metrics per increment in growth (1.0 z-score or 1.0 kg/m^2^ in BMI). Statistically significant values with p < 0.05 are in bold.

*Denotes p = 0.05–0.06.

### Neuroimaging markers and neurodevelopmental assessments

Pearson correlations comparing normalized global and regional brain imaging metrics to 4–6 year language outcomes found no statistically significant relationships. After controlling for all five covariates of interest, cortical thickness in the right temporal lobe was noted to be positively associated with PPVT4 score (parameter estimate 23.25, 95% CI 3.77 to 42.74).

### Neurodevelopmental assessments over time

In Pearson correlations of BSID-III language scores and 4–6 year language outcomes (Supplementary Table 5), PPVT4 and EVT2 were each positively related to language composite and both receptive and expressive scaled scores, with the most robust associations between EVT2 and language composite (r = 0.50, p = 0.002) and receptive scaled score (r = 0.56, p < 0.001). After controlling for sex, GA, highest parental education, and family income, the relationships between EVT2 and both BSID-III language composite and receptive language remained significant (Supplementary Table 5).

## Discussion

In this study investigating the relationship between growth in the NICU in EPT infants and their brain and language outcomes at early school age, we have provided the first evidence that growth anthropometrics at 36 weeks PMA are significantly associated with global brain volume and relative cortical thickness at 4 to 6 years of age in children with no known brain injury or neurologic abnormalities. These associations have potential implications for clinical practice and provide further evidence for the third trimester of gestation (experienced outside the womb for our participants) being a vital period of brain growth.

While no other study to our knowledge has related growth in the neonatal period to early school-age brain imaging, our findings are congruent with the work of other investigators who have previously evaluated the association of neonatal anthropometry with various neuroimaging metrics obtained at term equivalent age^10,12,52,53^. HC at term is strongly related to total brain volume at term^10,53^, and here we have shown that this relationship persists to school-age. In our unadjusted model, we also found that weight z-score at 36 weeks PMA to be positively associated with total intracranial volume at school age. Similarly, Coviello et al. demonstrated that weight gain in the first 4 weeks of life was correlated with total brain volume at term. In a study that included brain imaging in older school-age children, including those born both at term and preterm, Silva et al. tracked fetal and postnatal growth at multiple intervals, from 20 weeks gestation to 24 months of age, and found that greater weight gain during each age window was independently associated with larger brain volumes at 10 year of age^54^. Thus, achieving greater growth during the NICU hospitalization for preterm infants may have lasting effects in overall brain size.

Furthermore, we identified a novel negative association between HC at 36 weeks PMA and normalized cortical thickness in both hemispheres. While a negative relationship could be a sign of neuronal pruning, we did not observe the same findings using non-normalized metrics. We posit that this directionality instead could be explained by increases in global brain volume relative to the changes in cortical thickness. Although there were no significant findings relating growth and regional brain metrics in multivariable modeling, PPVT4 and right temporal cortical thickness were positively associated, consistent with our pilot study that previously reported on a subset of the patients in this cohort^24^.

Some studies have also examined long-term growth and neurodevelopment outcomes. Belfort et al. calculated the slopes of growth from birth to term equivalent age, from term to 4 months, and from 4 months to 1 year of age in an attempt to identify sensitive periods of postnatal growth. They concluded growth in the first two periods were predictive of higher BSID-II scores at 18 months^4^. In a separate cohort, improved linear growth from term to 4 months was more predictive of lower odds of IQ < 85^29^. Others have investigated relationships between growth in infancy and early childhood in very low birth weight and preterm children and have found some positive correlations between linear growth, weight gain, or increase in head circumference versus motor and cognitive outcomes at preschool and early school age^55,56^. There has been wide variation in the growth metrics related to outcome and the nature of the relationship, however. While we did not detect an association between NICU growth and language assessments at 4–6 years, for our sample, birth length z-score was positively associated with BSID-III language composite scores at 2 years corrected age. We also noted a strong association between length z-score at 36 weeks PMA and BSID-III language composite scores at 2 years, consistent with findings from Ramel et al.^6^, and we observed relationships between both composite and receptive language at 2 years and EVT2 scores at 4–6 years of age. Though it is unknown whether these results could be summative, it is possible that our small sample size prohibited us from discerning a direct relationship between growth and EVT2.

Although we were not able to obtain granular nutritional intake data, others have investigated nutrient intake and its association with specific brain imaging markers. In two different cohorts of preterm infants, patients who received increased macronutrient intakes had larger caudate size at term^57^ or at adolescence^58^. Early fat and caloric intake have been associated with regional brain volumetrics in preterm infants^12,13^, while another study found no relationship^59^. Protein and total energy intake via human milk specifically has also been related to greater total and regional brain volumes^60^. Again, these studies have primarily utilized term equivalent age brain imaging.

Taken together, our results and those reported by others suggest that optimizing intake and growth in the NICU has direct impact on the developing brain, including areas known to support language development. The cerebral hemispheres and cerebellum are undergoing a remarkable rate of growth during the period that children born EPT are hospitalized in the NICU^61,62^. Thus, providing the developing preterm brain with optimal nutritional substrate and closely monitoring and supporting somatic growth are logical targets for interventions aimed at decreasing morbidity from preterm birth. We speculate that these positive associations are due to increased growth of the neuronal cell populations in the cortex through infancy and childhood and not due to decreased pruning later in childhood. The children included in this report have not yet attained maximal cortical thickness (typically the peak occurs around 7 to 10 years for girls and 9 to 11 years for boys)^16,17^. Furthermore, other investigators have shown that, while EPT children in general have decreased intracranial volume, decreased grey and white matter volume, and decreased cortical surface area versus term children, the trajectory of cortical thinning is not significantly different between EPT children and term children^17,20,21,63–65^. Unfortunately, our findings in this report are consistent with those of other investigators in that the relationship between cortical morphometry and neurodevelopmental outcomes in EPT often do not survive multivariable adjustment, potentially due to limited sample size^19,21^. However, despite our relatively small sample size, these experiments did find an association between normalized cortical thickness in the right temporal region and language scores, which is congruent with a previous report from our group^24^. Future work could include an investigation of other markers of cortical morphometry, such as surface area and curvature, which have been shown to relate to language scores in preterm infants^66^.

Our study has a few relative limitations. We did not have body composition data for our participants, as this is a retrospective secondary analysis of a larger ongoing study. Newer studies evaluating fat-free (lean) mass have found associations with motor and cognitive outcomes at 1–2 years^7,8^ and brain size at term^11,52,67^. While we do include length and weight using standard growth curves, it remains unclear if weight or length is a better marker for lean mass and brain growth. Additionally, length and HC measurements are subject to error. At the time of the NICU hospitalizations of these infants, length boards were not routinely used. HC measurements could also be affected by inaccuracies related to delivery. Similarly, we did not prospectively assess nutritional intake, feeding composition, or feeding volumes in the NICU. However, given conflicting evidence, a robust relationship between intake in the NICU and brain growth (as indexed by volumetric analysis of MRI at term equivalent age) is still being established^12,13,59,60^. This could be due to difficulty reliably assessing enteral intake in the NICU, with some investigators finding nutritional intake in their studies failed to meet recommended amounts and others estimating enteral intake based on an assumed composition of maternal breast milk^12,13,59^, though accurate human milk analysis has been demonstrated to be capably incorporated in these studies^60^. Finally, we are relying on growth metrics assessed at birth and at 36 weeks PMA only. Future studies will increase the number of participants and the granularity of data collection, enabling a better assessment of nonlinear variables such as growth curves through higher-order techniques, such as functional data analysis.

In summary, we have reported significant positive associations between neonatal growth in the NICU and total and regional brain volumes at early school age for children born extremely preterm. Somatic growth in the NICU did not correlate with language scores at school-age for this cohort. Our hypothesis-generating pilot study results suggest that growth in the NICU has significant and lasting effects on brain development in children born EPT and that this is an exciting avenue for future research.

### Supplementary Information

Supplementary Tables.

## Data Availability

Data used in the manuscript are available to editors upon request to the corresponding author.
