# Supplementary material for: Correlation of NICU anthropometry in extremely preterm infants with brain development and language scores at early school age
Source: Sci Rep. 2023 Sep 15;13:15273. doi: 10.1038/s41598-023-42281-0 (PMC10504298; doi:10.1038/s41598-023-42281-0)
Supplement: Supplementary file 1 — Supplementary Tables. [file 41598_2023_42281_MOESM1_ESM.pdf]

Supplemental Table 1 – Univariate analyses of covariates of interest with neurodevelopmental and brain MRI metrics

	**BSID-III Composite Scores at 2 Years**			**Assessment Scores at 4-6 years**			**Global Brain Metrics**			**Regional Brain Metrics**						
	Cognitive	Language	Motor	PPVT4	EVT2	WNV	eTIV (mm^3)	Cortical Thickness (Left) (mm)	Cortical Thickness (Right) (mm)	Volume: Corpus Callosum (mm^3)	Volume: Cerebellum (Left) (mm^3)	Volume: Cerebellum (Right) (mm^3)	Volume: Temporal (Left) (mm^3)	Volume: Temporal (Right) (mm^3)	Cortical Thickness: Temporal (Left) (mm)	Cortical Thickness: Temporal (Right) (mm)
Gestational Age at Birth (weeks)	3.3 (-2.0, 8.5)	3.3 (-1.8, 8.3)	1.5 (-2.1, 5.2)	1.7 (-1.4, 4.8)	1.1 (-1.6, 3.7)	1.1 (-2.6, 4.9)	-12147.5 (-43741.7, 19446.7)	0.03 (-0.02, 0.09)	0.03 (-0.02, 0.08)	**104.5 (17.0, 191.9)**	674.4 (-461.8, 1810.6)	678.2 (-534.3, 1890.8)	204.1 (-504.9, 913.1)	111.8 (-602.0, 825.5)	0.03 (-0.02, 0.09)	0.04 (-0.02, 0.09)
Sex	7.2 (-2.9, 17.3)	**13.3 # (4.2, 22.3)**	5.9 (-1.0, 12.8)	0.5 (-6.5, 7.5)	2.8 (-3.0, 8.6)	-5.1 (-13.3, 3.1)	-62944.6 (-132503.0, 6613.8)	**0.14 (0.02, 0.25)**	**0.14 (0.03, 0.24)**	124.3 (-88.5, 337.0)	2291.5 (-240.9, 4823.9)	2062.5 (-672.5, 4797.5)	106.3 (-1525.0, 1737.6)	364.6 (-1267.1, 1996.3)	**0.13 (0.01, 0.25)**	**0.13 (0.01, 0.26)**
Family Income	**12.4 (2.1, 22.6)**	**11.3 (1.3, 21.4)**	7.1 * (-0.1, 14.3)	**13.7 # (7.5, 19.8)**	**6.7 (0.7, 12.7)**	0.8 (-8.3, 10.0)	29723.9 (-45928.5, 105376.4)	0.01 (-0.12, 0.14)	0 (-0.12, 0.13)	55.8 (-178.5, 290.0)	2487.9 (-258.2, 5234.0)	2288.6 (-673.8, 5250.9)	325.7 (-1402.0, 2053.4)	1225.0 (-416.3, 2866.3)	-0.01 (-0.15, 0.12)	0.01 (-0.13, 0.16)
Parental Education: Some College	10.8 (-4.5, 26.2)	9.3 (-4.6, 23.2)	2.4 (-8.4, 13.3)	0 (-11.3, 11.4)	6.5 (-2.1, 15.1)	5.7 (-7.5, 18.8)	32998.2 (-80762.4, 146758.9)	-0.10 (-0.30, 0.09)	-0.10 (-0.29, 0.09)	25.3 (-314.8, 365.4)	-1149.7 (-5299.4, 3000.0)	-1463.0 (-5877.4, 2951.5)	-792.1 (-3451.4, 1867.1)	1154.6 (-1454.9, 3764.1)	-0.09 (-0.29, 0.11)	-0.07 (-0.29, 0.14)
Parental Education: College	5.0 (-12.7, 22.7)	**17.3 (1.3, 33.4)**	8.7 (-3.9, 21.2)	1.7 (-10.7, 14.0)	**12.4 (2.9, 21.9)**	13.1 (-1.3, 27.5)	84544.5 (-37070.7, 206159.7)	-0.11 (-0.32, 0.10)	-0.11 (-0.31, 0.08)	-2.7 (-366.3, 360.9)	655.3 (-3780.8, 5091.5)	-849.9 (-5569.2, 3869.3)	-1253.5 (-4096.3, 1589.4)	1159.0 (-1630.7, 3948.7)	-0.12 (-0.33, 0.10)	-0.09 (-0.32, 0.14)
Parental Education: Some Graduate	0.8 (-20.9, 22.6)	1.2 (-18.5, 20.8)	0.5 (-14.9, 15.9)	-11.3 (-27.1, 4.6)	4.8 (-7.6, 17.3)	5.0 (-13.8, 23.8)	-20750.8 (-172432.0, 130930.1)	-0.07 (-0.33, 0.20)	-0.05 (-0.30, 0.20)	-42.6 (-496.1, 410.8)	-3289.7 (-8822.5, 2243.2)	-4068.7 (-9954.6, 1817.2)	-934.5 (-4480.2, 2611.2)	-606.6 (-4086.0, 2872.7)	-0.03 (-0.30, 0.24)	-0.03 (-0.32, 0.25)
Parental Education: Graduate Level	14.5 (-1.4, 30.4)	**19.5 # (5.1, 33.9)**	6.7 (-4.5, 17.9)	5.6 (-6.1, 17.3)	**12.1 # (3.1, 21)**	7.2 (-6.3, 20.7)	-534.7 (-118941.0, 117871.1)	0.01 (-0.20, 0.21)	0 (-0.19, 0.19)	232.8 (-121.2, 586.8)	850.9 (-3468.2, 5170.0)	760.9 (-3833.7, 5355.6)	-1095.6 (-3863.5, 1672.2)	1232.9 (-1483.2, 3948.9)	-0.01 (-0.22, 0.20)	0.04 (-0.18, 0.26)
Corrected Age at MRI (years)	3.2 (-2.9, 9.3)	-1.6 (-7.7, 4.4)	1.7 (-2.5, 6.0)	-0.3 (-4.4, 3.8)	-1.7 (-5.1, 1.7)	-1.0 (-5.9, 3.8)	-18630.3 (-60757.4, 23496.8)	0.01 (-0.06, 0.08)	0.01 (-0.06, 0.08)	-8.3 (-135.1, 118.5)	-337.5 (-1885.1, 1210.2)	-12.6 (-1665.2, 1640.0)	-629.8 (-1556.6, 297.0)	-239.7 (-1191.9, 712.5)	0 (-0.07, 0.07)	0.01 (-0.07, 0.09)

All brain metrics normalized for eTIV.

Parameter estimates (95% CI) represent change in outcome metrics per increment in age or relative to reference category (sex: male; income: less than $50,000; highest parental education: high school).

Statistically significant parameter estimates with $p<0.05$ are in **bold**; # denotes $p<0.01$; * denotes $p=0.05-0.06$.

Supplemental Table 2 – Covariate selection for each multivariable regression model

	**Gestational Age at Birth**	**Sex**	**Family Income or Insurance Status**	**Highest Parental Education**	**Corrected Age at MRI**
**Growth vs. BSID-III**	x	x	x	x	
**Growth vs. PPVT4/EVT2/WNV**	x	x	x	x	
**Growth vs. Brain MRI**	x	x	x	x	x
**Brain MRI vs. PPVT4/EVT2/WNV**	x	x	x	x	x
**BSID-III vs. PPVT4/EVT2/WNV**	x	x	x	x	

Family insurance status and highest parental education obtained at time of BSID-III (2 year) testing.
Family income and highest parental education obtained at time of PPVT4/EVT2/WNV (4-6 year) testing.
BSID-III and PPVT4/EVT2/WNV assessments take into account the exact age at time of testing.

Supplemental Table 3 – Pearson correlations comparing growth with brain MRI metrics (non-normalized)

		Global Brain Metrics		Regional Brain Metrics						
		Cortical Thickness (Left) (mm)	Cortical Thickness (Right) (mm)	Volume: Corpus Callosum (mm^3)	Volume: Cerebellum (Left) (mm^3)	Volume: Cerebellum (Right) (mm^3)	Volume: Temporal (Left) (mm^3)	Volume: Temporal (Right) (mm^3)	Cortical Thickness: Temporal (Left) (mm)	Cortical Thickness: Temporal (Right) (mm)
**Birth**	Weight z-score	-0.11	-0.14	0.11	-0.03	-0.06	0.33	**0.36**	0.08	0.07
	Length z-score	0.11	0.09	0.00	-0.05	-0.06	**0.35**	**0.36**	0.21	0.29
	HC z-score	0.03	0.05	0.21	0.12	0.12	**0.36**	**0.36**	0.19	0.13
	BMI	-0.20	-0.19	0.32	0.04	0.04	0.25	0.29	0.02	-0.03
**36 Weeks PMA**	Weight z-score	-0.11	-0.02	0.15	0.01	0.01	**0.46 #**	**0.37**	0.20	0.12
	Length z-score	0.15	0.18	0.07	0.04	0.00	**0.38**	0.33	**0.43**	0.24
	HC z-score	-0.02	0.13	0.12	0.19	0.17	**0.47 #**	**0.49 #**	0.29	0.21
	BMI	-0.25	-0.17	0.15	-0.02	0.02	0.21	0.14	-0.12	-0.08
**Change from Birth to 36 Weeks PMA**	Weight z-score	0.01	0.13	0.03	0.05	0.08	0.11	-0.02	0.12	0.04
	Length z-score	0.04	0.09	0.08	0.08	0.06	0.06	0.00	0.24	-0.02
	HC z-score	-0.05	0.06	-0.13	0.04	0.02	0.02	0.04	0.04	0.05
	BMI	-0.16	-0.08	0.01	-0.04	0.00	0.10	0.01	-0.13	-0.07

Statistically significant Pearson r correlation coefficients with $p<0.05$ are in **bold**; # denotes $p <0.01$.

Supplemental Table 4 – Multivariable regression model comparing growth with brain MRI metrics (non-normalized)

		Global Brain Metrics		Regional Brain Metrics						
		Cortical Thickness (Left) (mm)	Cortical Thickness (Right) (mm)	Volume: Corpus Callosum (mm^3)	Volume: Cerebellum (Left) (mm^3)	Volume: Cerebellum (Right) (mm^3)	Volume: Temporal (Left) (mm^3)	Volume: Temporal (Right) (mm^3)	Cortical Thickness: Temporal (Left) (mm)	Cortical Thickness: Temporal (Right) (mm)
**Birth**	Weight z-score	0.01 (-0.04, 0.06)	0.004 (-0.05, 0.06)	251.9 (-24.3, 528.1)	-3059.4 (-7426.6, 1307.7)	-3079.0 (-7717.0, 1559.0)	667.0 (-1379.1, 2713.1)	374.6 (-2335.4, 3084.7)	0.04 (-0.01, 0.09)	0.04 (-0.06, 0.13)
	Length z-score	0.02 (-0.02, 0.07)	0.02 (-0.03, 0.07)	77.2 (-207.1, 361.5)	-3504.1 (-7658.1, 650.0)	-3486.4 (-7911.6, 938.8)	771.6 (-1201.1, 2744.2)	456.2 (-2164.5, 3076.8)	0.03 (-0.02, 0.09)	0.07 (-0.01, 0.16)
	HC z-score	0.02 (-0.02, 0.06)	0.03 (-0.01, 0.07)	**232.0 (16.0, 448.1)**	-175.3 (-3820.0, 3469.3)	-154.1 (-4009.4, 3701.3)	602.2 (-1032.8, 2237.3)	567.8 (-1593.9, 2729.5)	**0.04 (0.001, 0.08)**	0.03 (-0.04, 0.11)
	BMI	-0.005 (-0.05, 0.04)	-0.01 (-0.06, 0.04)	238.9 (-20.0, 497.7)	-191.3 (-4461.8, 4079.1)	-85.8 (-4603.5, 4431.8)	534.1 (-1391.3, 2459.4)	758.1 (-1770.2, 3286.3)	0.02 (-0.03, 0.07)	-0.01 (-0.09, 0.08)
**36 Weeks PMA**	Weight z-score	0.02 (-0.03, 0.07)	0.03 (-0.02, 0.08)	194.7 (-71.3, 460.7)	-16.2 (-4298.3, 4265.9)	357.4 (-4169.4, 4884.3)	1651.5 (-165.0, 3468.0)	1722.4 (-728.6, 4173.4)	**0.05 (0.005, 0.1)**	0.06 (-0.02, 0.15)
	Length z-score	-0.004 (-0.05, 0.04)	-0.001 (-0.05, 0.05)	50.0 (-215.4, 315.4)	-2157.3 (-6155.4, 1840.8)	-2009.6 (-6262.6, 2243.4)	889.1 (-933.3, 2711.4)	870.6 (-1547.2, 3288.3)	0.04 (-0.01, 0.08)	0.04 (-0.05, 0.12)
	HC z-score	0.001 (-0.04, 0.05)	0.03 (-0.02, 0.074)	114.0 (-148.9, 376.9)	1650.7 (-2403.8, 5705.2)	1815.3 (-2468.2, 6098.9)	**1933.4 (250.9, 3615.8)**	**2640.1 (448.6, 4831.6)**	0.04 (-0.002, 0.09)	0.06 (-0.02, 0.14)
	BMI	0.01 (-0.01, 0.03)	0.02 (-0.005, 0.04)	106.5 (-20.3, 233.3)	918.5 (-1113.9, 2950.9)	1084.1 (-1056.2, 3224.4)	556.0 (-353.4, 1465.4)	579.3 (-630.5, 1789.2)	0.01 (-0.01, 0.04)	0.02 (-0.03, 0.06)
**Change from Birth to 36 Weeks PMA**	Weight z-score	0.01 (-0.04, 0.06)	0.03 (-0.02, 0.07)	-27.2 (-299.4, 244.9)	2582.1 (-1469.6, 6633.8)	2956.9 (-1302.5, 7216.3)	1017.1 (-837.5, 2871.8)	1333.3 (-1105.1, 3771.8)	0.02 (-0.03, 0.07)	0.03 (-0.06, 0.12)
	Length z-score	-0.03 (-0.08, 0.02)	-0.03 (-0.08, 0.03)	-22.3 (-328.9, 284.3)	1163.7 (-3533.1, 5860.5)	1339.4 (-3623.7, 6302.5)	293.4 (-1845.9, 2432.7)	631.3 (-2173.2, 3435.8)	0.01 (-0.05, 0.07)	-0.04 (-0.13, 0.06)
	HC z-score	-0.02 (-0.06, 0.02)	-0.003 (-0.04, 0.04)	-134.3 (-357.3, 88.7)	1388.3 (-2103.8, 4880.4)	1490.1 (-2202.0, 5182.3)	864.4 (-700.9, 2429.7)	1420.5 (-606.1, 3447.2)	-0.01 (-0.05, 0.04)	0.02 (-0.06, 0.09)
	BMI	0.01 (-0.01, 0.03)	0.01 (-0.01, 0.03)	39.1 (-78.0, 156.2)	746.1 (-1047.2, 2539.3)	855.2 (-1036.6, 2747.0)	333.6 (-480.5, 1147.7)	311.0 (-767.0, 1389.0)	0.01 (-0.02, 0.03)	0.01 (-0.02, 0.05)

Adjusted for sex, gestational age, highest parental education, family income, age at MRI.
Parameter estimates (95% CI) represent change in brain MRI metrics per increment in growth (1.0 z-score or 1.0 kg/m^2 in BMI).
Statistically significant parameter estimates listed with $p<0.05$; NS = not significant

Supplemental Table 5 – Comparison of BSID-III scores at 2 years and assessment scores at 4-6 years

		**BSID-III**						
		Cognitive Composite Score	Language Composite Score	Language: Expressive Scaled	Language: Receptive Scaled	Motor Composite Score	Motor: Gross Scaled	Motor: Fine Scaled
**Pearson r Correlation**	PPVT4	0.26	**0.37**	**0.34**	**0.38**	0.33 *	0.23	0.29
	EVT2	0.30	**0.50 #**	**0.39**	**0.56 #**	0.23	0.11	0.25
	WNV	0.00	0.11	-0.03	0.24	0.10	0.00	0.19
**Multivariable Parameter Estimate**	PPVT4	-0.02 (-0.29, 0.25)	0.25 (-0.03, 0.54)	1.14 (-0.25, 2.53)	1.50 (-0.23, 3.22)	0.21 (-0.14, 0.56)	1.26 (-0.61, 3.13)	0.62 (-0.93, 2.16)
	EVT2	0.11 (-0.11, 0.34)	**0.26 (0.02, 0.5)**	0.93 (-0.30, 2.16)	**1.84 (0.42, 3.25)**	0.07 (-0.24, 0.39)	-0.15 (-1.85, 1.55)	0.67 (-0.69, 2.03)
	WNV	0.02 (-0.32, 0.35)	0.07 (-0.30, 0.44)	-0.07 (-1.89, 1.75)	0.90 (-1.32, 3.11)	0.09 (-0.36, 0.53)	-0.23 (-2.63, 2.18)	0.91 (-1.02, 2.84)

Multivariable model adjusted for sex, gestational age, highest parental education (at 4-6 years), and family income (at 4-6 years).
Parameter estimates (95% CI) represent change in 4-6 year assessment scores per increment in BSID-III scores.
Statistically significant Pearson r correlation coefficients and parameter estimates with $p<0.05$ are in **bold**; # denotes $p <0.01$; * denotes p=0.05-0.06.
